# The In Situ Structure of T-Series T1 Reveals a Conserved Lambda-Like Tail Tip

**DOI:** 10.3390/v17030351

**Published:** 2025-02-28

**Authors:** Yuan Chen, Hao Xiao, Junquan Zhou, Zeng Peng, Yuning Peng, Jingdong Song, Jing Zheng, Hongrong Liu

**Affiliations:** 1 Key Laboratory for Matter Microstructure and Function of Hunan Province, School of Physics and Electronics, Hunan Normal University, Changsha 410082, China; 2 Institute of Interdisciplinary Studies, Hunan Normal University, Changsha 410082, China; 3 National Institute of Pathogen Biology, Chinese Academy of Medical Sciences & Peking Union Medical College, Beijing 102629, China; 4 The National & Local Joint Engineering Laboratory of Animal Peptide Drug Development, College of Life Sciences, Hunan Normal University, Changsha 410081, China

**Keywords:** T-series phages, lambda-like siphophages, cone-shaped tail tip, disulfide bond, cryo-EM

## Abstract

It is estimated that over 60% of known tailed phages are siphophages, which are characterized by a long, flexible, and non-contractile tail. Nevertheless, entire high-resolution structures of siphophages remain scarce. Using cryo-EM, we resolved the structures of T-series siphophage T1, encompassing its head, connector complex, tail tube, and tail tip, at near-atomic resolution. The density maps enabled us to build the atomic models for the majority of T1 proteins. The T1 head comprises 415 copies of the major capsid protein gp47, arranged into an icosahedron with a triangulation number of seven, decorated with 80 homologous trimers and 60 heterotrimers along the threefold and quasi-threefold axes of the icosahedron. The T1 connector complex is composed of two dodecamers (a portal and an adaptor) and two hexamers (a stopper and a tail terminator). The flexible tail tube comprises approximately 34 hexameric rings of tail tube. The extensive disulfide bond network along the successive tail rings may mediate the flexible bending. The distal tip of T1, which is cone-shaped and assembled by proteins gp33, gp34, gp36, gp37, and gp38, displays structural similarity to that of phage lambda. In conjunction with previous studies of lambda-like siphophages, our structure will facilitate further exploration of the structural and mechanistic aspects of lambda-like siphophages.

## 1. Introduction

Siphophages possess a long, flexible, and non-contractile tail that plays a crucial role in host recognition, cell envelope penetration, and DNA injection [1,2]. Connected to the head through a connector complex, the tail of a siphophage consists of three main components: a tail tube, a tape measure protein (TMP), and a distal tip [3,4]. During infection, the tail tip penetrates the cell envelope. Triggering TMP release transmits a signal along the tail tube to the head, enabling DNA release into the host cytoplasm [2,5,6]. All siphophages resolved at near-atomic resolution exhibit common morphological features, including the head, the connector complex, and the tail tube [7,8,9,10]. Nevertheless, their tail tips exhibit remarkable structural diversity, with examples including the tips of siphophages lambda [7], R4C [9], 80α [11], TP901-1 [12], and JBD30 [13]. Siphophages with a cone-shaped distal tip are widely distributed in nature. A representative example is siphophage lambda [7,14], which has a tip that mainly contains the distal tail protein, hub protein, central fiber protein, and insertion protein, flanked with lateral fibers. Nevertheless, structural characterization of lambda-like siphophages remains limited. To date, only four siphophage structures have been resolved at high resolution, namely lambda [7], T5 [15], DT57C [8], and Chi [16]. During the infection process, the central fiber proteins of lambda [17] and T5 [18,19] are capable of bending and bringing the tail closer to the cell membrane, thereby facilitating further infection. In contrast, the tail tip of DT57C does not exhibit this bending behavior [8]. Despite these siphophages sharing characteristic cone-shaped distal tips, their specific structural details require further elucidation.

Siphophage T1 is representative of the T series of model *Escherichia coli* phages, which has been observed to infect not only sensitive strains of *E. coli* but also some strains of Shigella dysenteriae [20,21]. Initial interest in T1 was prompted by its remarkable capacity to contaminate stocks of other phages or cultures of *E. coli* in laboratory [22]. The present research on T1 has focused on three primary areas: the assembly of its head [23], the identification of its membrane receptor proteins, FhuA [24], and the replication and transcription of its DNA following its entry into infected host cells [22,25,26]. Notably, phages typically employ a variety of strategies to facilitate their effective propagation, which can be classified into lysogenic and lytic life cycles [27]. T1, a typical lytic siphophage, has been shown to exist in a novel lysogenic state, and it is hypothesized that this prophage state further integrates information about the density of bacterial populations and the metabolic state of the host, thereby controlling its transition from a lysogenic to a lytic cycle [20]. The T1-spanin gene is synthesized into target bacteria and incorporated into non-proliferative phages using phage-based technology. These non-proliferative phages have been shown to exhibit strong antibacterial activity against the various Gram-negative bacteria, which is important for the future development of innovative antimicrobial agents [28,29]. Despite a series of biochemical studies on T1, the lack of atomic structures further hinders our in-depth understanding of T1.

Using cryo-EM, we resolved the three-dimensional (3D) structures of the icosahedral head, head–tail complex and the tail tip of a laboratory-adapted fiberless mature T1 at resolutions of 3.5 Å, 3.6 Å, and 4.3 Å, respectively. These structures enabled us to build atomic models for the head proteins (gp47, gp48 and gp49), the connector complex proteins (gp42, gp44, gp45 and gp52), the tail tube protein (TTP) gp41, and the tail tip proteins (gp33, gp34, gp36, gp37 and gp38). Notably, the flexible tail tube of T1 may be mediated by the disulfide bonds between two adjacent tail rings, which allows for more efficient capture of host receptors. The structure of the cone-shaped distal tail tip of T1 is highly similar to that of phage lambda [7]. In addition, the entire head–tail structure of T1 is also conserved compared with the widespread lambda-like members. The results of this study offer insights into the infection process and evolution of the siphophages with a cone-shaped distal tip.

## 2. Materials and Methods

### 2.1. Production and Purification of Siphophages T1

*E. coli* strain ATCC 11303 was cultivated in Luria–Bertani broth (yeast extract, 5 g; tryptone, 10 g; and NaCl, 10 g/L) at 37 °C for 24 h. T1 phages (ATCC 11303-B1) were next incubated with the bacterial cell for 15 min at 37 °C. Then, the mixture was propagated in 1 L of the bacteria cell for 4 h at 37 °C. Subsequently, the cell debris was separated through centrifugation at 6000× *g* for 20 min at 8 °C. Phage particles in the supernatant were gathered with 1M NaCl and 10% polyethylene glycol (PEG) 8000 (Amresco, Solon, OH, USA) by overnight at 4 °C, followed by centrifugation at 11,000× *g* for 1h at 4 °C. The precipitated phages were resuspended using phage buffer (50 mM NaCl, 10 mM MgCl_2_, 10 mM Tris-HCl, and pH 7.6) (Amresco, Solon, OH, USA), and then were purified on 1.7 g/mL, 1.5 g/mL and 1.45 g/mL CsCl cushions (Sigma, St. Louis, MO, USA) through ultracentrifugation at 130,000× *g* for 2 h at 8 °C. The T1 phage band was collected and dialyzed in phage buffer at 4 °C overnight. Finally, purified T1 phages were stored in ice-water for cryo-sample processing.

### 2.2. Data Acquisition and Image Processing

The purified phage T1 aliquot (3.5 µL) was pipetted onto graphene oxide (GO) grids, which were glow-discharged for 30 s. The grids were then loaded into a Thermo Fisher Scientific (TFS) Vitrobot (Waltham, MA, USA), with humidity of 100%, and blotted for 3.5 s at 8 °C. After blotting out the excess solution on the grids, the grids were vitrified using liquid ethane and transferred to liquid nitrogen. The data were collected using a Thermo Scientific Krios G3i transmission electron microscope (Waltham, MA, USA) equipped with a K3 direct electron detector (Gatan, Pleasanton, CA, USA) at a magnification of 53,000×, resulting in a calibrated size of 1.36 Å/pixel. The total dose for each movie was approximately 32 e^−^/Å^2^. Finally, a total of 3319 images were collected, with each movie stack comprising 32 image frames. The astigmatism and defocus values of each image were calculated using GCTF [30] packaged in RELION 3.1.4 software (Cambridge CB2 0QH, UK) [31]. Using ETHAN-1.9 software [32], a total of 112,442 particle images were boxed. Based on the common-line algorithm [33,34], the icosahedral head of T1 was reconstructed to a resolution of 4 Å using our programs [35,36]. The fivefold region of the icosahedral head was then improved to a resolution of 3.5 Å, using the local reconstruction method [37,38].

### 2.3. Symmetry-Mismatch and Local Reconstruction of the Head–Connector Complex

The asymmetric structure of siphophage lambda, filtered to a resolution of 60 Å, was used as an initial model [7]. Using the symmetry-mismatch reconstruction method [39], the 3D asymmetric structure of the head–connector complex in T1 was reconstructed to a resolution of 8 Å. The fundamental process was as follows. (1) For each 2D particle imagine, the asymmetrical orientation was searched by the 60 equivalent orientations of the icosahedral orientation based on an initial model from lambda. (2) Using the latest orientation, a new asymmetric structure of the head–connector complex in T1 was reconstructed, without imposing any symmetry. (3) The above two processes were iterated in approximately 70 rounds until the resolution of the asymmetric structure could not be improved any further. Using the local reconstruction method [37,38], we next improved the structure of the portal–adaptor complex and stopper–terminator–tube complex of T1 to resolutions of 3.6 and 3.6 Å by imposing twelvefold and sixfold symmetry, respectively.

### 2.4. Local Reconstructions of the Tail Tube and Tail Tip

Using the RELION 3.1.4 software [40], the local structures of the tail tip and tail tube of T1 particles were reconstructed. With the aid of the latest orientation of the asymmetric structure of the head–connector complex in T1 described above, a new asymmetric structure of the head–connector complex was obtained using RELION 3.1.4 software. Subsequently, we focused the center on the tail tube with a box size of 240 × 240 pixels. A total of 90,576 tail tube particles were boxed using EMAN 1.9 Helix software [41]. 2D and 3D classification were next performed. After excluding overly curved tail tube structures, 39,589 particles from the 2D classifications were selected for the 3D classification. Finally, the tail tube was reconstructed to a resolution of 4.1 Å by imposing sixfold symmetry.

A total of 27,652 tail tip particles were manually selected with a box size of 240 × 240 pixels. After excluding non-tip and other irrelevant structures, we selected 10,726 particles from 2D classifications to perform 3D classification by imposing threefold symmetry. We next selected the suited three types (a total of 7216 particles) for 3D classification to further correct the center for refinement. The structure of the tail tip was resolved to a resolution of 6.1 Å by imposing threefold symmetry. Subsequently, using the local refinement and reconstruction method [37,38], we selected 4467 particles based on the correlation coefficient and reconstructed the tail tip to a resolution of 4.3 Å by imposing threefold symmetry.

### 2.5. Atomic Modeling Building and Refinement

Based on our cryo-EM density maps of T1, the atomic models of MCP gp47, N-terminus of CP gp48, CP gp49, portal protein gp52, adaptor protein gp45, stopper protein gp44, tail terminator protein gp42, TTP gp41, distal tail protein gp37, hub protein gp36, N-terminus of central fiber protein gp33, insertion protein gp34, and C-terminus of TMP gp38 were manually built using COOT 0.9.8.1 software [42]. All atomic models were further refined through real-space refinement, implemented in Phenix 1.20.1 [43]. The refinement and validation statistics are shown in Table S1.

### 2.6. Overall Structure of Phage T1

Mature T1 phage was purified from *E. coli* strain ATCC 11303 for cryo-EM data acquisition (Figure S1A). A total of 112,442 mature particles were extracted from 3319 micrographs. Using icosahedral reconstruction [35], we initially obtained an icosahedral head structure of the mature T1 at a resolution of 4 Å. Subsequently, using the local reconstruction method [37,38], we reconstructed the fivefold region of the icosahedral head to a resolution of 3.5 Å (Figure S1B). Using symmetry-mismatch reconstruction method [39], we determined an asymmetric structure of the head–connector complex of T1 at 8 Å resolution. By employing the local refinement and reconstruction method [37,38], we then resolved the portal–adaptor complex and stopper–terminator–tube complex of T1 to resolutions of 3.6 and 3.6 Å by imposing twelvefold and sixfold symmetry, respectively (Figure S1B). Using RELION 3.1.4 software [40], we selected the tail tube and tail tip from the micrographs and obtained their structures to resolutions of 4.1 and 6.1 Å, respectively (Figure S1B). Finally, using the local refinement and reconstruction method [37,38], we resolved the tail tip to a resolution of 4.3 Å (Figure S1B). Local resolution maps for the T1 from the head to the tip are presented in Figure S2, generated using ResMap-1.1.4 [44]. The high-resolution maps allowed us to build the atomic models for the major capsid protein (MCP) gp47, cement protein (CP) gp49, N-terminus of CP gp48, portal protein gp52, adaptor protein gp45, stopper protein gp44, tail terminator protein gp42, and TTP gp41 (Figure S3A,B). Furthermore, the atomic model of the distal tail protein gp37, hub protein gp36, N-terminus of the central fiber protein gp33, insertion protein gp34, and C-terminus of TMP gp38 were also obtained (Figure S3B). However, the C-terminus of CP gp48, the N-terminus of TMP, and the C-terminus of the central fiber protein gp33 could not be resolved, likely due to their flexibility. The resolution of the structures was estimated using a cutoff of 0.143 based on the gold-standard Fourier shell correlation curve [45].

**Figure 1:**
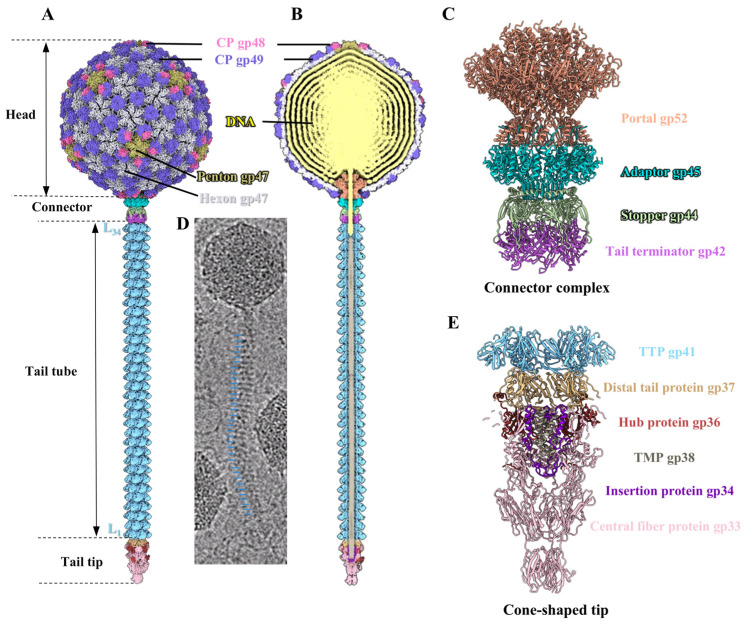
Overall structure of siphophage T1. (**A**,**B**) Side (**A**) and cut-open (**B**) views of the asymmetric structure of T1. L_1_ and L_34_ denote the initial (first) and final (thirty-fourth) layers of the tail tube, respectively. The color code is applied to panels (**A**–**C**,**E**). (**C**) Side view of the ribbon models of connector complex. (**D**) Zoomed-in view of a particle image of T1. The possible TTP rings are labeled by blue lines. (**E**) Cut-open view of the ribbon models of the cone-shaped tail tip and a tail tube ring.

The T1 head comprises 415 copies of the MCP gp47 with a triangulation number (T) of seven (Figure 1A). Additionally, 80 trimeric CPs gp49 and 60 heterotrimeric CPs (each comprising one gp48 monomer and two gp49 monomers) are attached to the surface of the head along the icosahedral threefold and quasi-threefold axis, respectively (Figure 1A). This further enhances the head stability. The connector complex, which is connected to a unique fivefold vertex of the head, comprises two dodecamers (one portal and one adaptor) and two hexamers (one stopper and one tail terminator) (Figure 1B,C). The tail tube is stacked by approximately 34 hexameric rings of the TTP gp41 (Figure 1B,D). The channel of the tail tube is filled with trimeric TMP gp38 and the distal tail is connected to a cone-shaped tip, which contains the hexameric distal tail protein gp37, threefold hub protein gp36, threefold central fiber protein gp33, and threefold insertion protein gp34 (Figure 1E). Table S1 presents the details of the data collection and reconstruction process.

### 2.7. Structure of the Head

**Figure 2:**
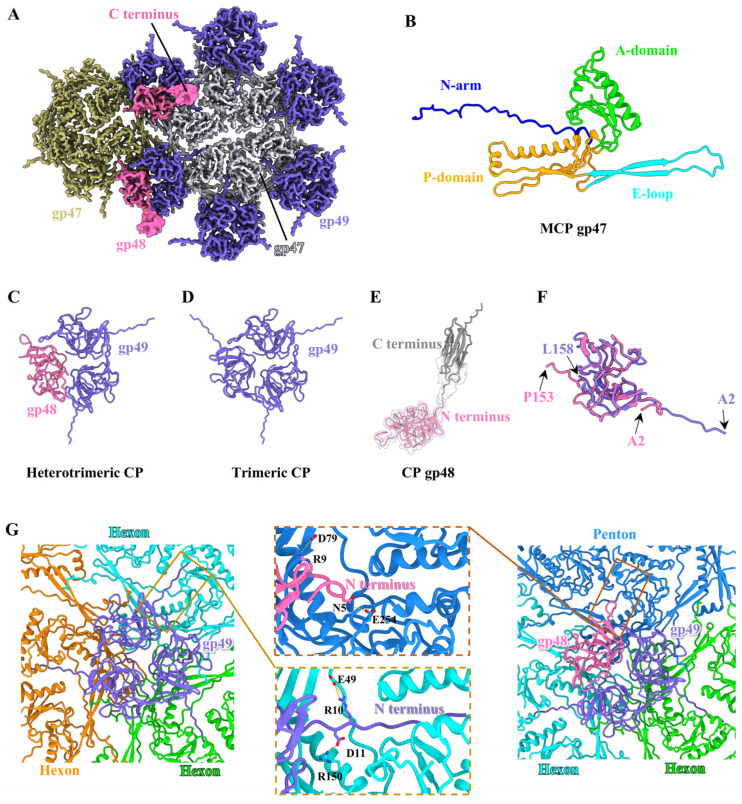
Structures of MCPs and CPs of T1. (**A**) Density maps of a hexon (light steel blue), a penton (olive), and six trimeric CPs comprising four homotrimers and two heterotrimers. The CP gp48 and gp49 are colored in hot pink and slate blue, respectively. (**B**) Ribbon model of the MCP gp47 shown in four domains. (**C**,**D**) Top view of the ribbon models of the heterotrimeric CP (**C**) and homotrimeric CP (**D**). (**E**) Density map (transparency) of CP gp48 superimposed on the ribbon model and C-terminus of gp48 modeled using AlphaFold3. (**F**) Structural comparison between gp49 (slate blue) and the N-terminus of gp48 (pink) revealing the obvious topological similarity. (**G**) Interactions at the capsomere–CP interface around the threefold axis (left) and quasi-threefold axis (right) of the icosahedral head. The insets show the zoomed-in views of the structural difference of the N-terminus between gp48 and gp49 interacting with the capsomeres.

Based on the density map of the T1 head, we built the atomic model of MCP gp47 from residues 27 to 319 (out of 319 residues) (Figure 2A and Figure S3B), which displays a structural similarity to the MCP gp5 in HK97 [46] (Figure S4A), MCP gp7 in VP1 [47] and MCP gp2 in GP4 [48]. According to the nomenclature of the MCP gp5 in HK97, each gp47 is composed of a long N-terminal arm (residues 27–61), a periphery P-domain (residues 62–70, 108–166 and 275–306), an extended E-loop (residue 71–107), and an axis A-domain (residues 167–274 and 307–319) (Figure 2B). An asymmetric unit of the icosahedral head comprises seven gp47 monomers, including one hexon and one-fifth of a penton (Figure S4B). The structural superimposition of the seven gp47 monomers in an asymmetric unit reveals significant conformational change in the E-loops and N-arms (Figure S4C). The conformational discrepancy of all gp47 monomers permits the formation of 11 pentons and 60 hexons, which are ultimately assembled into an icosahedral capsid with T = 7 (Figure S4D). It is noteworthy that the two types of CP are situated around the icosahedral threefold and quasi-threefold axes of the T1 head (Figure 2A), including homologous trimeric CP gp49 and heterotrimeric CP (each comprising one gp48 monomer and two gp49 monomers) (Figure 2C,D). We built the atomic models for gp49 from residues 2 to 158 of the total 158 residues and for the N-terminus of gp48 from residues 2 to 153 (out of the 255 residues) (Figure S3B). However, the C-terminus of gp48, exhibited a weak protrusion density, which could not be resolved to a high resolution (Figure 2A), and its atomic model (residues 154–255) was generated in conjunction with AlphaFold3 (Figure 2E). Both gp49 and the N-terminus of gp48 adopt a typical β-tulip fold structure (Figure 2C,D), which is analogous to that observed in the CP gp56 of TW1 [49], and CP gpD of lambda [50] and triplex proteins of herpesvirus [51]. The structural comparison of the two types of CPs reveals that gp49 in the heterotrimeric CPs adopts a topology identical to that in the homologous trimeric CPs (Figure S4E). However, gp49 exhibits a slight conformational difference from the N-terminus of gp48 (Figure 2F).

The inter-capsomer and capsomer–CP interfaces of T1 exhibit extensive and complex interactions, including the salt bridges, hydrogen bonds, and electrostatic interactions. The N-terminus of MCP gp47 from a capsomere interacts with the P-domain of MCP gp47 from the adjacent capsomer via the salt bridge along the twofold axis (Figure S5A,B). Additionally, the P-domains and E-loops from neighboring capsomers around the threefold axis are linked by the salt bridges, hydrogen bonds, and electrostatic interactions (Figure S5C–E). Furthermore, it is intriguing that the extended N-terminus of gp49 from the two types of CPs is directly embedded in the inner surface of the head and forms close contact with the A-domain of two adjacent MCPs (Figure 2G). This interaction model differs from those of previous structural studies [48,50,52], in which phages encode dimeric or trimeric CPs that are merely anchored to the outer surface to reinforce the capsid stability. Although gp48 of heterotrimeric CPs is also merely attached to the outer surface of the head (Figure 2G), the outward protrusion of its C-terminus may assist T1 adsorption on the host in the early stage of infection, as previously described in T5 [15] and φcrAss001 [53].

### 2.8. Structure of the Connector Complex

**Figure 3:**
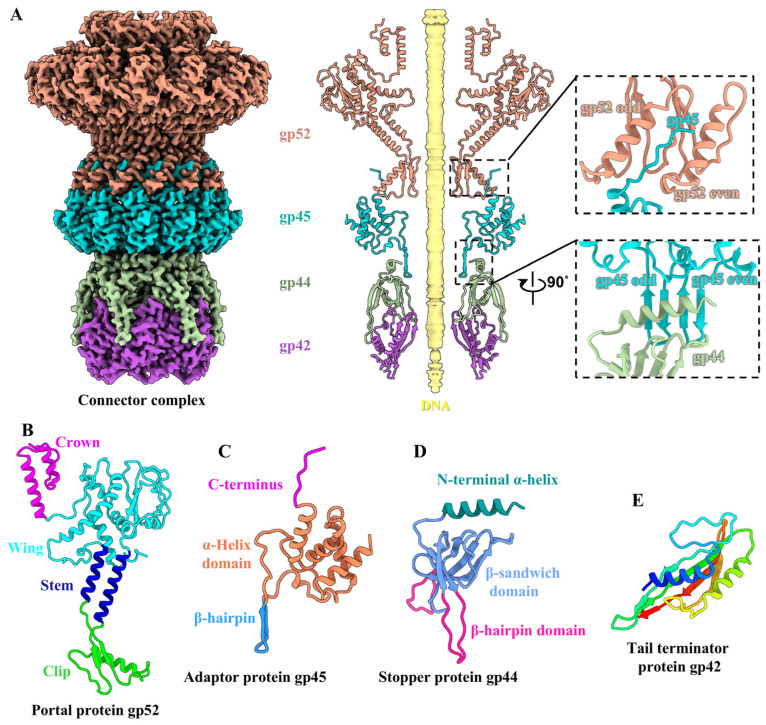
Structures of the connector complex. (**A**) Side view (left) of the density maps and slab view (right) of the ribbon models of the connector complex. The insets show the zoomed-in views of the interactions among the portal–adaptor and the adaptor–stopper. The color coding is identical to that used in Figure 1A. (**B**–**D**) Ribbon models of the portal protein gp52 (**B**), the adaptor protein gp45 (**C**), and the stopper protein gp44 (**D**), colored according to their domains. (**E**) Ribbon model of the tail terminator protein gp42 shown in rainbow colors.

The T1 connector complex, which serves as a channel for DNA delivery during infection, is composed of a dodecameric portal, a dodecameric adaptor, a hexameric stopper, and a hexameric tail terminator (Figure 3A). In addition, the structure of all connector proteins of T1 is highly similar to that of siphophage lambda [7] (Figure S6A). The T1 portal comprises 12 copies of protein gp52, which exhibits structural conservation across the majority of tailed phages [54]. Each gp52 can be divided into four domains: the crown domain (residues 357–405), the wing domain (residues 32–192 and 291–356), the stem domain (residues 193–215 and 275–290) and the clip domain (residues 216–274) (Figure 3B). The T1 adaptor is composed of 12 copies of protein gp45. Each gp45 is composed of an α-helix domain (residues 2–80 and 94–121), a β-hairpin domain (residues 81–93), and an extended C-terminus (residues 122–131) (Figure 3C), which displays a structural similarity to the adaptor proteins of gp81 and gp82 of phage GP4 [55], protein p144 of phage T5 [15], and protein gp15 of SPP1 [56]. The C-terminus of gp45 extends into the interaction interfaces between the clip domain of two adjacent portal proteins gp52 (Figure 3A). Additionally, the 12 copies of the β-hairpin domain of the adaptor form a 24-stranded β-barrel structure, which serves as a docking site for the stopper (Figure 3A). The T1 stopper contains six copies of protein gp44, each of which comprises three domains: an α-helix domain surrounding the β-hairpin domain of the adaptor (residues 1–17) (Figure 3A), a β-sandwich domain (residues 18–29, 45–57, and 72–123), and a β-hairpin domain with two extended β-strands (residues 30–44 and 58–71) (Figure 3D). However, only partial siphophages [9,56] and myophages [57,58] encode a hexameric stopper, which is believed to be responsible for the transition of the connector complex from twelvefold symmetry to sixfold symmetry. The T1 tail terminator is constituted by six copies of protein gp42 (Figure 3E), which exhibit structural homology to those observed in siphophages [8,9,16], myophages [57,58], and contractile injection systems (CISs) [59,60]. The tail terminator is attached to the tail tube, terminating the growth of the tail tube [1] (Figure 1B). Complementary electrostatic potentials are observed among the pairwise interfaces between the protein components of the T1 connector complex (Figure S6B).

### 2.9. Structure of the Tail Tube

**Figure 4:**
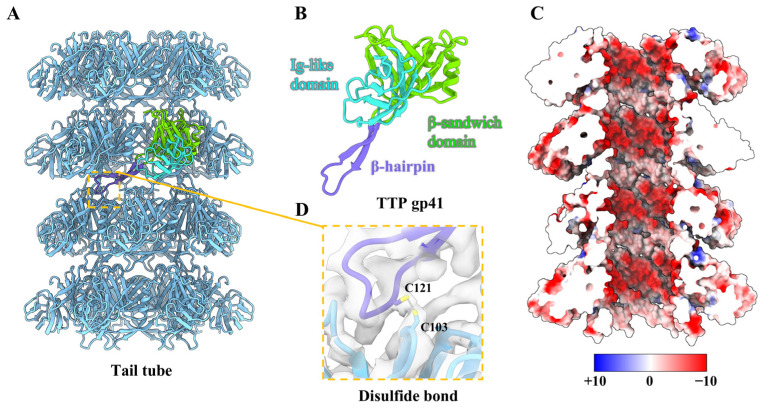
Structure of the tail tube. (**A**) Side view of four layers of hexameric tail tube (cornflower blue). Only one monomer of tail tube protein gp41 is colored according to its domains. (**B**) Ribbon model of TTP gp41, colored according to its domains. (**C**) Cut-open view of the electrostatic potential of the inner surfaces of tail tube. The electrostatic potential scale is shown in the color bar. (**D**) Zoomed-in view of the inter-ring interactions by the disulfide bond.

The T1 tail tube is composed of approximately 34 hexamer rings of TTP gp41, which collectively form an extended right-handed helix structure with a helical rise of ~42 Å and a twist of ~27° (Figure 4A and Figure S7A,B). The lumen of the tail tube is filled with TMP gp38 (Figure 1B). Each protein gp41 contains three domains: a β-sandwich domain (residues 2–15, 95–113 and 135–218), an immunoglobulin-like (Ig-like) domain (residues 16–94), and a β-hairpin domain (residues 114–134) (Figure 4B). The β-sandwich domain constitutes the main body of the tail tube, exhibiting the topological identity to that observed in siphophages [7,56], myophages [58,61] and CISs [59,60]. Six copies of the β-sandwich domain of gp41 oligomerize to form a continuous 24-stranded β-barrel hexamer, which mediates the intra-ring interactions (Figure 4A). Each TTP monomer is decorated with an additional Ig-like domain, which extends outside from the β-sandwich domain and forms a conserved Ig-like fold (Figure 4B). Notably, the Ig-like domain of the outer surface of the tail tubes among reported siphophages displays a substantial structural diversity [62], such as gp22 in YSD1 [10], gpV in lambda [7], gp44 in JBD30 [13] and gp22 in Chi [16] (Figure S7C). Additionally, the central channel of the tail tube exhibits a strong negative electrostatic potential, which may facilitate the ejection of DNA into the host cell, as observed in other TTPs, such as R4C [9], lambda [7], and YSD1 [10] (Figure 4C).

It has been hypothesized that the interspace and distance between successive rings, in conjunction with the extended flexible β-hairpin domain, are key factors contributing to the flexibility of the tails in siphophages. This allows them to withstand mechanical forces without breaking [2,62]. Notably, the β-hairpin domain of gp41 from one ring closely interacts with the β-sandwich domain of gp41 in the adjacent ring by forming a disulfide bond, which may mediate the inter-ring interaction in the flexible tail tube of T1 (Figure 4D). The sequence alignment of TTPs between T1 and other reported siphophages [7,11,13,16] has shown that all TTPs exhibit low sequence identities (~10%) (Figure S8) and no conserved disulfide bond, which mediates the inter-ring interaction, is found in these TTPs. This disulfide-bond feature has only been previously observed in the myophage Milano [63], which contains a curved tail with the tube and sheath regions compared to other myophages. In addition to the inherent characteristics that influence the bending of the tail tube, we hypothesize that the formation of disulfide bonds at the molecular level may facilitate the compression or stretching of TTP in T1 and Milano within the tail tube during the initial stages of infection. Previous structural analysis has revealed that the tail tube does not directly participate in infection or signal transduction, but primarily serves a scaffolding function [64,65]. The flexible structure of the tail tubes may therefore facilitate a more efficient screening of host receptors for siphophages.

### 2.10. Structure of the Tail Tip

**Figure 5:**
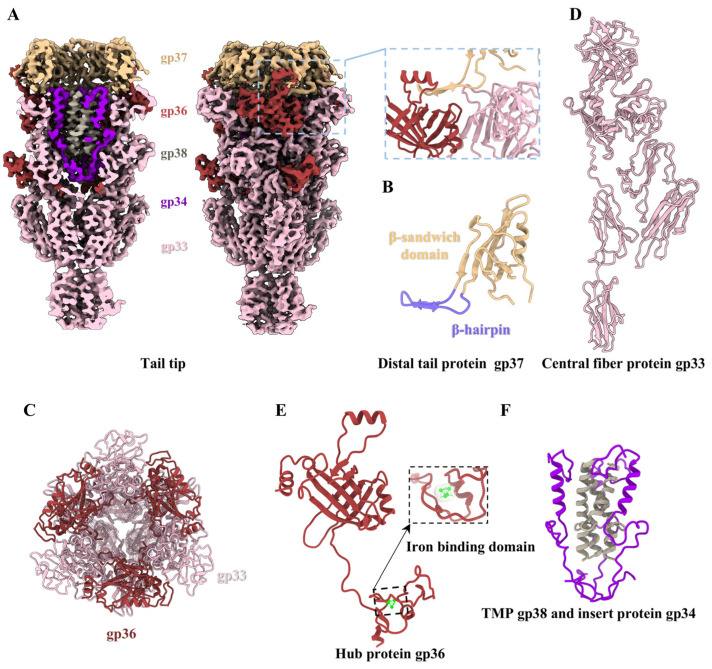
Structure of the tail tip. (**A**) Cut-open (left) and side (right) views of the density maps of the tail tip without the lateral fibers. The inset shows a zoomed-in view of the interactions among the distal tail protein gp37, center fiber protein gp33, and hub protein 36. The color coding is identical to that used in Figure 1A. (**B**) Ribbon model of the distal tail protein gp37, colored according to its domains. (**C**) Top view of the interactions between the threefold central fiber protein gp33 and the threefold hub protein gp36. (**D**) Ribbon models of the center fiber protein gp33. (**E**) Ribbon models of hub protein 36 and zoomed-in view of the iron–sulfur cluster (green) superimposed on its density map (transparent). (**F**) Interactions between the trimeric TMP gp38 (gray) and the threefold insert protein gp34 (blue violet).

**Figure 6:**
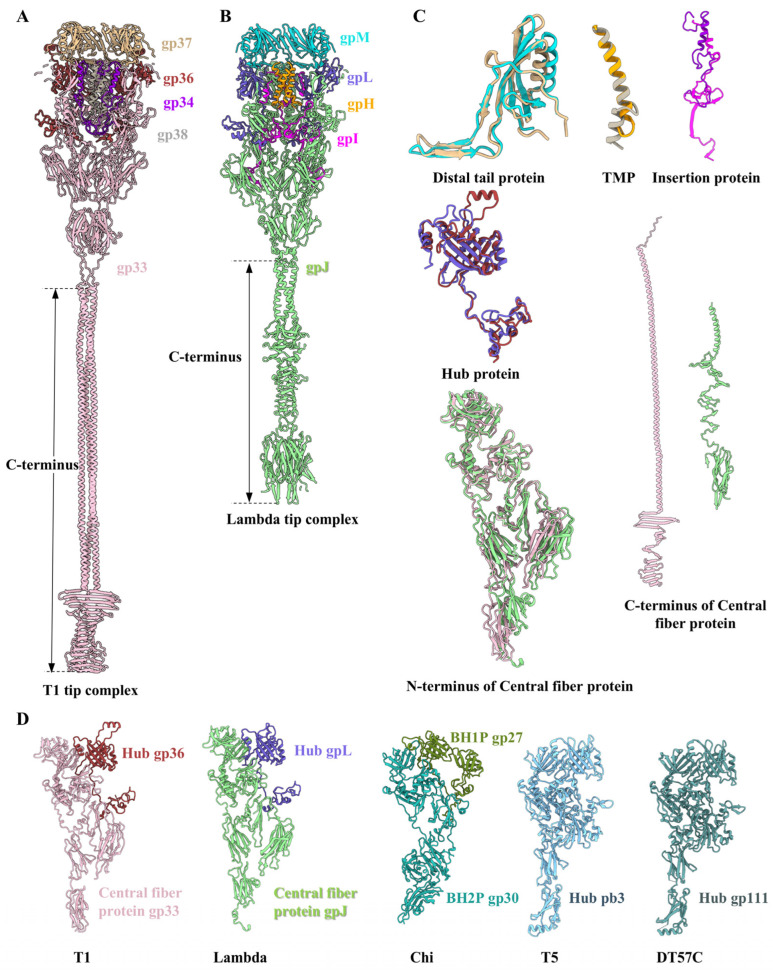
Structural comparison of tail tip proteins in different siphophages. (**A**,**B**) Cut-open views of ribbon models of the tail tips in T1 (**A**) and lambda ((**B**), PDB ID: 8k35 and 8xck). (**C**) Structural comparisons of ribbon models of all tail tip proteins between T1 and lambda, revealing the obvious topological similarity. The color coding is identical to that used in (**A**,**B**). (**D**) Structural comparisons of ribbon models of the tip (the central fiber protein and/or hub protein) among lambda-like siphophages, including T1, lambda (PDB ID: 8k35 and 8xck), Chi (PDB ID: 8VJH), T5 (PDB ID: 7zhj), and DT57C (PDB ID: 8hqz).

The tail tip is situated at the distal end of the tail, comprising the hexameric distal tail protein gp37, the threefold hub protein gp36, the trimeric central fiber protein gp33, and the trimeric insertion protein gp34 (Figure 5). Similarly, as observed in laboratory-adapted siphophages lambda [7] and T5 [15], no density map for the lateral tail fibers was identified in the laboratory-adapted T1 (Figure 1A and Figure 5A). Despite the differences in detailed interactions, the arrangements and structural features of the all proteins in the T1 tail tip are similar to those described for lambda without the lateral fibers [7] (Figure 6A–C).

As in the structures of the distal tail proteins in siphophages lambda [7], Chi [16], and T5 [15], each gp37 in T1 contains a β-sandwich fold domain (residues 2–24 and 45–117) and a β-hairpin domain (residues 25–44) (Figure 5B). Additionally, gp37 exhibits a similar TTP-like fold, but lacks the Ig-like domain (Figure S7C). Six copies of the β-sandwich fold domain of gp37 form a hexameric ring that interacts with the extended β-hairpin domains of the first TTP ring (Figure 1E). The β-hairpin domains of the distal tail protein gp37 are bonded to the top of the threefold hub protein and threefold central fiber protein, thereby mediating the transformation from sixfold symmetry to threefold symmetry (Figure 5A).

The central fiber protein gp33 is coupled with the hub protein gp36, which together seal the distal end of the tail, resembling an inverted cone (Figure 5A,C). The extension region of the tail tip is exclusively formed by gp33 (Figure 6A). The trimeric central fiber protein gp33 is the largest protein in the tail tip (Figure 5D). We built an atomic model of the N-terminus of protein gp33 from residues 13–820 (out of the 1172 residues) (Figure S3B). Its C-terminus (residue 821–1172) forms the farthest distal structure, but a density map could not be obtained due to the ultrahigh flexibility of this protein (Figure 5A). According to AlphaFold3 predictions, the C-terminus of the central fiber protein gp33 in T1, which is visibly longer than that of gpJ in lambda [7], protrudes approximately 370 Å from the tail tip and forms an α/β-helix fold structure (Figure 6A,B and Figure S9). This analogous structural feature of spikes or fibers in the distal tip is a common occurrence in phages for the purposes of host recognition or/and membrane perforation [66]. We modeled 242 residues (16–257) of the 260-residue gp36 protein (Figure 5E). Its N-terminal α-helix extends upwards and interacts with the distal tail protein gp37 (Figure 5A). In particular, its C-terminal density is obviously clustered (Figure 5E). Therefore, we suspect that T1 gp36 may show functional and structural similarly to the gpL from lambda [7,67] in that its C-terminus forms an iron-binding domain, which interacts with the iron-sulfur cluster to assist in stabilizing the hub protein during its assembly and to facilitate further modification of the tail tip. Notably, the central fiber protein and hub protein of T1 and lambda, as well as the baseplate hub 1 protein (BH1P) and baseplate hub 2 protein (BH2P) of Chi [16], polymerize into a trimeric complex, displaying a topological similarity to the baseplate hub protein pb3 of T5 [15] and the tail tip hub protein gp111 of DT57C [8] (Figure 6D).

The C-terminus of the TMP gp38 and the insertion protein gp34 were observed to be located within the lumen of the inverted cone (Figure 5A). TMP in siphophages and myophages plays a crucial role in interacting with chaperone proteins to initiate TTP polymerization and in determining the length of the tail [68,69]. We only obtained the density map of the C-terminus (residues 921–956) of TMP gp38 (Figure 5F), and the remainder of gp38 could not be resolved, probably because of its inherent flexibility or asymmetric assembly in the tail tube. We also built an atomic model of protein gp34 from residues 92–163 (out of the 199 residues) (Figure S3B). The trimeric insertion protein gp34 oligomerizes into a tripod to support the coiled coil of the trimeric TMP in the tip lumen (Figure 5F), with each comprising a flexible long loop followed by a C-terminal helix (Figure 5F). This similar structural feature was also identified in siphophages lambda [7] and Chi [16]. However, there is no structural homologue of the insertion protein in the tip of the related siphophage T5 [15] and DT57C [8], possibly due to the differences in their environment and host cells. Despite the absence of significant sequence homology, the structural association among these tip proteins is intimate, suggesting a potential common evolutionary origin and reflecting the extensive evolutionary history of these lambda-like siphophages with the inverted cone-shaped tip.

## 3. Discussion

We have resolved the entire structure of the T1 at near-atomic resolution by cryo-EM. The density maps enable us to build the atomic models for the MCP gp47 and CPs (gp48 and gp49), connector complex (portal gp52, adaptor gp45, stopper gp44, and tail terminator gp42), and TTP gp41. In combination with alphaFold3, we also obtained all atomic models of the tail tip, including the distal tail protein gp37, the hub protein gp36, the central fiber protein gp33, the insertion protein gp34, and the C-terminus of TMP gp38. Notably, besides the interspace and distance between successive rings and the β-hairpin domain that contribute to the tail flexibility of siphophages, the disulfide bond network along the entire tail tube may also mediate the flexibility of the T1 tail. The flexibility of the tail may enable the receptor-binding protein to explore larger spaces for the host receptor, resulting in enhancing their competition and adaptation to hostile environments. This also confirms that T1 has been shown to be remarkably destructive in the laboratory.

The comparative analysis of the structural characteristics among T1 and siphophages reveals that the majority of protein components from the head to the tail in T1 exhibit structural similarities with those of reported siphophages, which suggests that they share common evolutionary origins. Nevertheless, the tail tips of these siphophages exhibit significant variation in their structural features [5,6,13]. In particular, the features of the T1 tail tip, which are essential for infection, trans-membrane and DNA ejection, are structurally conserved across the widespread lambda-like siphophages with the cone-shaped tip, thus suggesting that T1 is another representative of lambda-like siphophages. Furthermore, structural comparisons of the tail tip among T5 [15], DT57C [8], Chi [16], lambda [7] and T1 reveal that with the exception of the conserved and essential structural components of the inverted tip formed by the distal tail protein, hub, and central fiber protein, etc., a variety of add-on components diversify the inverted tip decorated with different lateral fibers, receptor-binding proteins or collars, etc. (Table S2). For instance, the distal tip of T5 and DT57C transform from a triple bifurcation to an extended β-helix structure [8,15], while the distal tip of T1 forms a long and flexible α/β-helix fold structure. In contrast, Chi does not encode an extended fiber or spike to modify the distal tip [16]. During the infection process, the binding of the phages T5 [18] and lambda [17] to the receptor on the envelope of *E. coli* is accompanied by structural reorganization of the tail tip, where the distal tip bend and bring the tail closer to the cell membrane. The structural differences of the distal tip in DT57C may allow it to directly infect the host cell without bending [8]. However, whether the tip of T1 is curved during the infection still needs to be further studied. The structural similarities and differences observed among these lambda-like siphophages likely derive from their adaptation to diverse environments and their ability to adsorb differently on various host cells, further confirming the long evolutionary history of siphophages.

## Data Availability

Cryo-EM maps and the structures have been deposited at the Electron Microscopy Data Bank (EMD-62664, EMD-62682, EMD-62698, EMD-62669 and EMD-62912) and the Protein Data Bank (PDB ID: 9KZJ, 9L01, 9L0E, 9L0F and 9L9P) and are publicly available as of the date of publication.

## Supplementary Materials

The following supporting information can be downloaded at https://www.mdpi.com/article/10.3390/v17030351/s1. Figure S1: Cryo-EM images and FSC curves; Figure S2: Local resolution maps; Figure S3: Quality of density maps and atomic models; Figure S4: Structures of the MCP and CP monomers of T1; Figure S5: The interactions of the capsomere-capsomere; Figure S6: The structure and electrostatic potential of the connector complex; Figure S7: Structural comparison of the different tail tubes; Figure S8: Sequence alignment of the TTPs; Figure S9: The pLDDT and PAE of T1 gp33; Table S1: Reconstruction and refinement statistics; Table S2: The structural similarities and differences in siphophage tips.
