# Supplementary material for: The In Situ Structure of T-Series T1 Reveals a Conserved Lambda-Like Tail Tip
Source: Viruses. 2025 Feb 28;17(3):351. doi: 10.3390/v17030351 (PMC11945409; doi:10.3390/v17030351)
Supplement: Supplementary file 1 [file viruses-17-00351-s001.zip › viruses-3448648-supplementary.pdf]

A

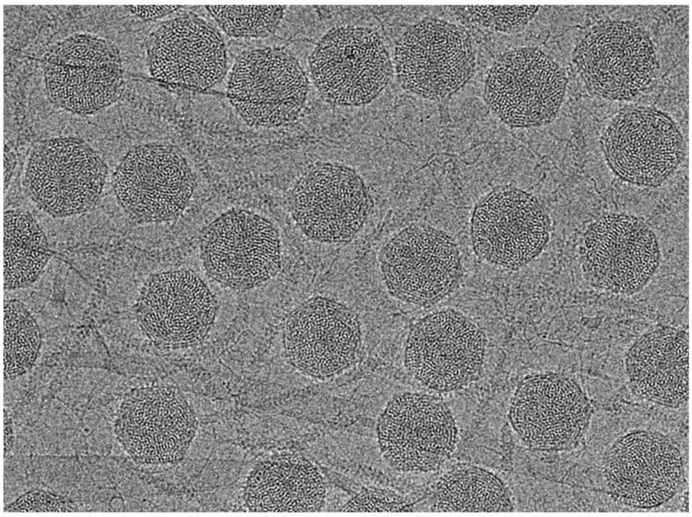

B

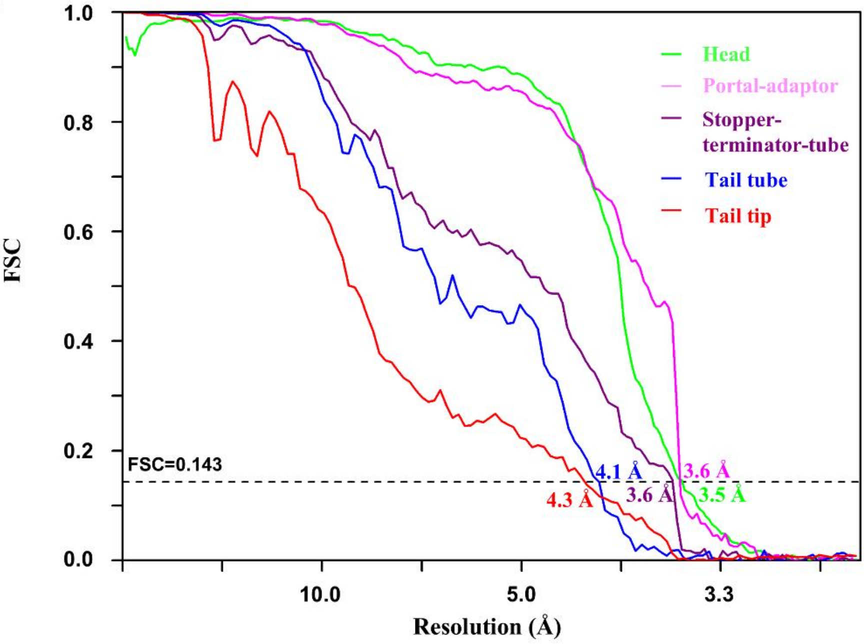

Figure S1. Cryo-EM images and Fourier shell correlation curves of siphophage T1. (A) Representative cryo-EM image of T1. (B) Estimated structural resolutions of the five-fold region of the icosahedral head (green line), portal-adaptor (pink line), stopper-terminator-tube (purple line), tail tube (blue line) and tail tip (red line) of T1.

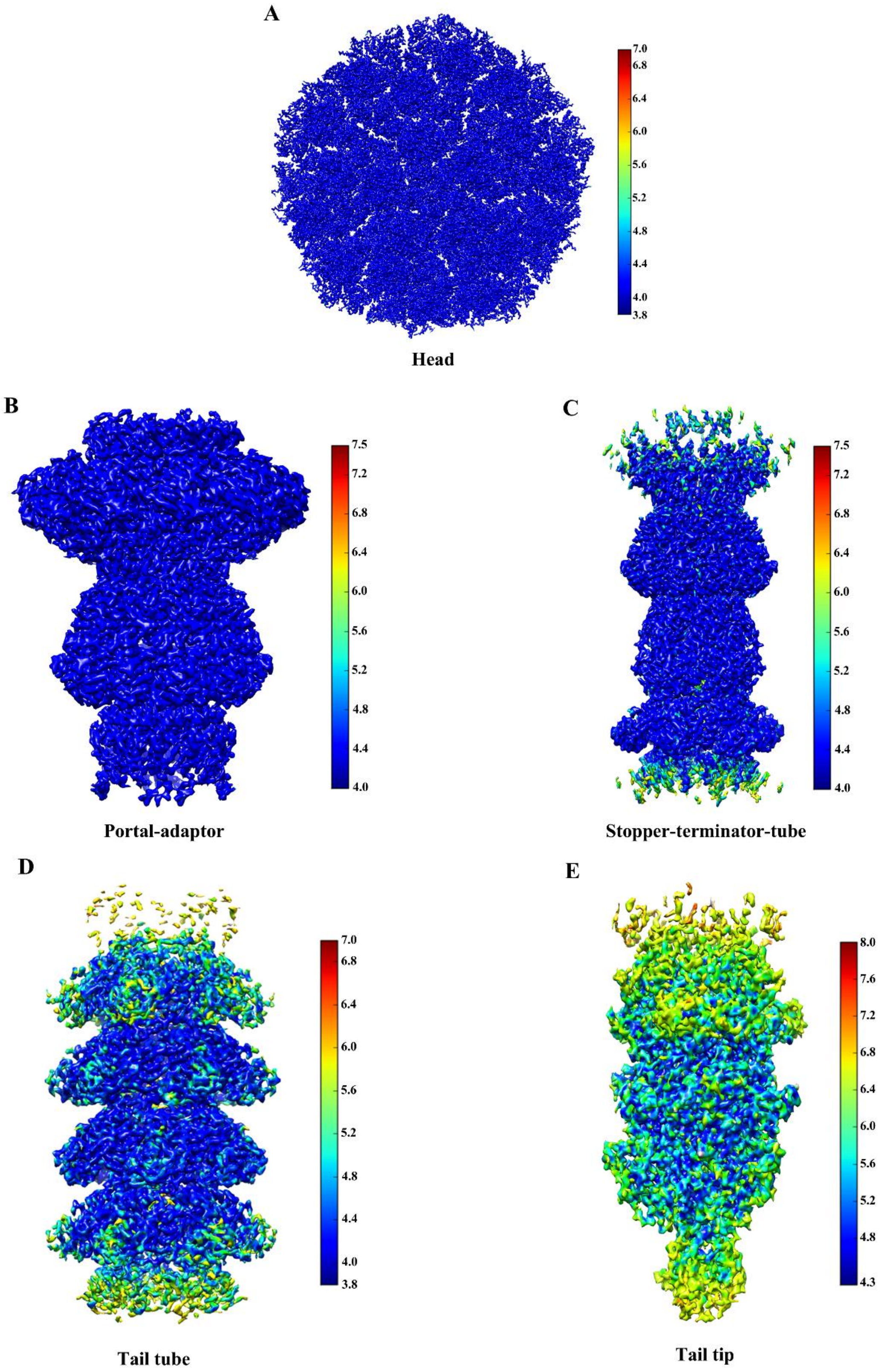

Figure S2. Local resolution maps of the T1 from the head to the tip.

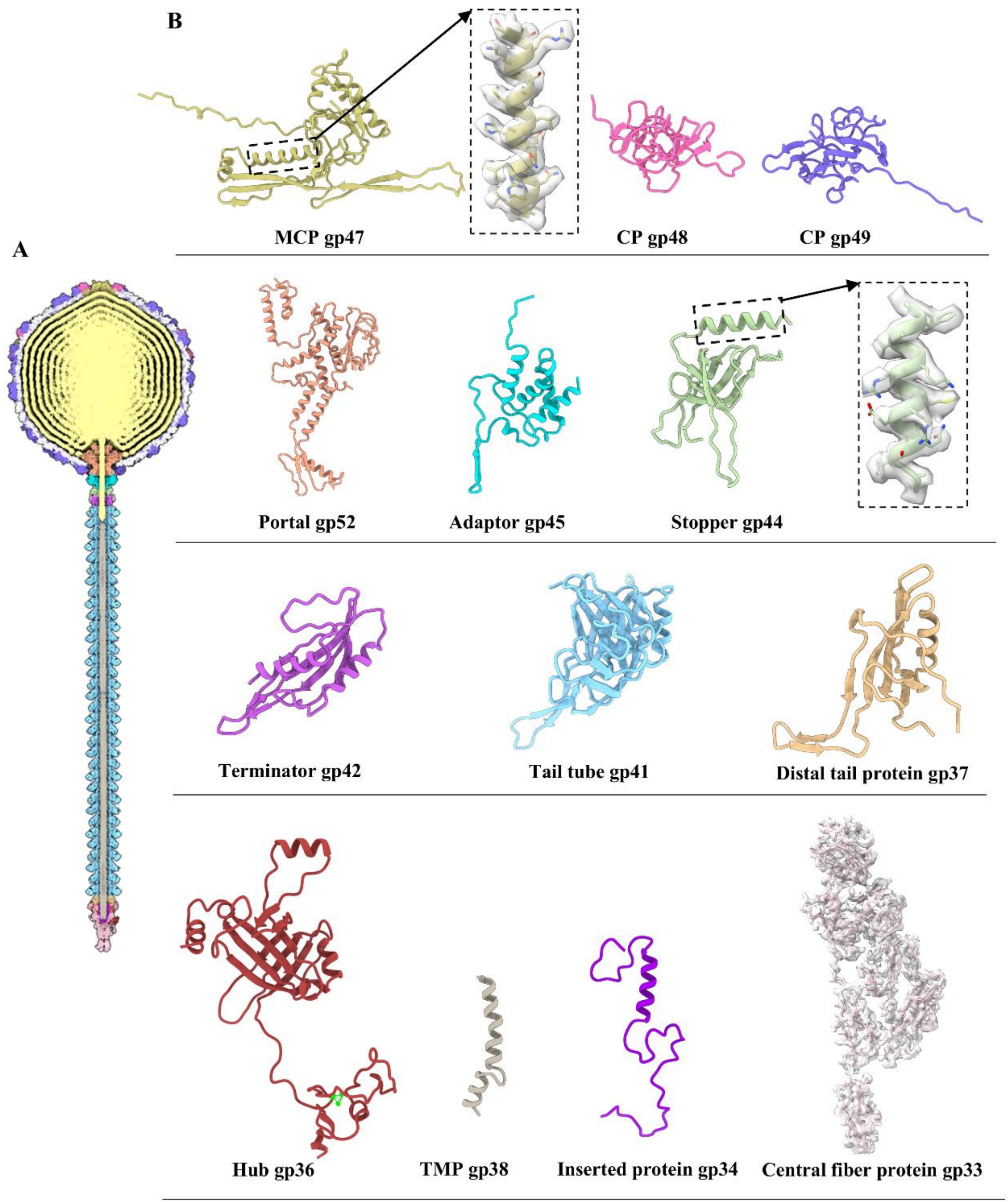

Figure S3. Quality of the cryo-EM density maps and the atomic models of the 13 proteins of T1. (A) Cut-open view of the asymmetric structure of T1. All color codes are identical to those used in Fig. 1A. (B) Ribbon models of 13 proteins from the head to the tail tip. The insets show the zoomed-in views of density maps (transparency) superimposed on the atomic models of partial proteins (sticks).

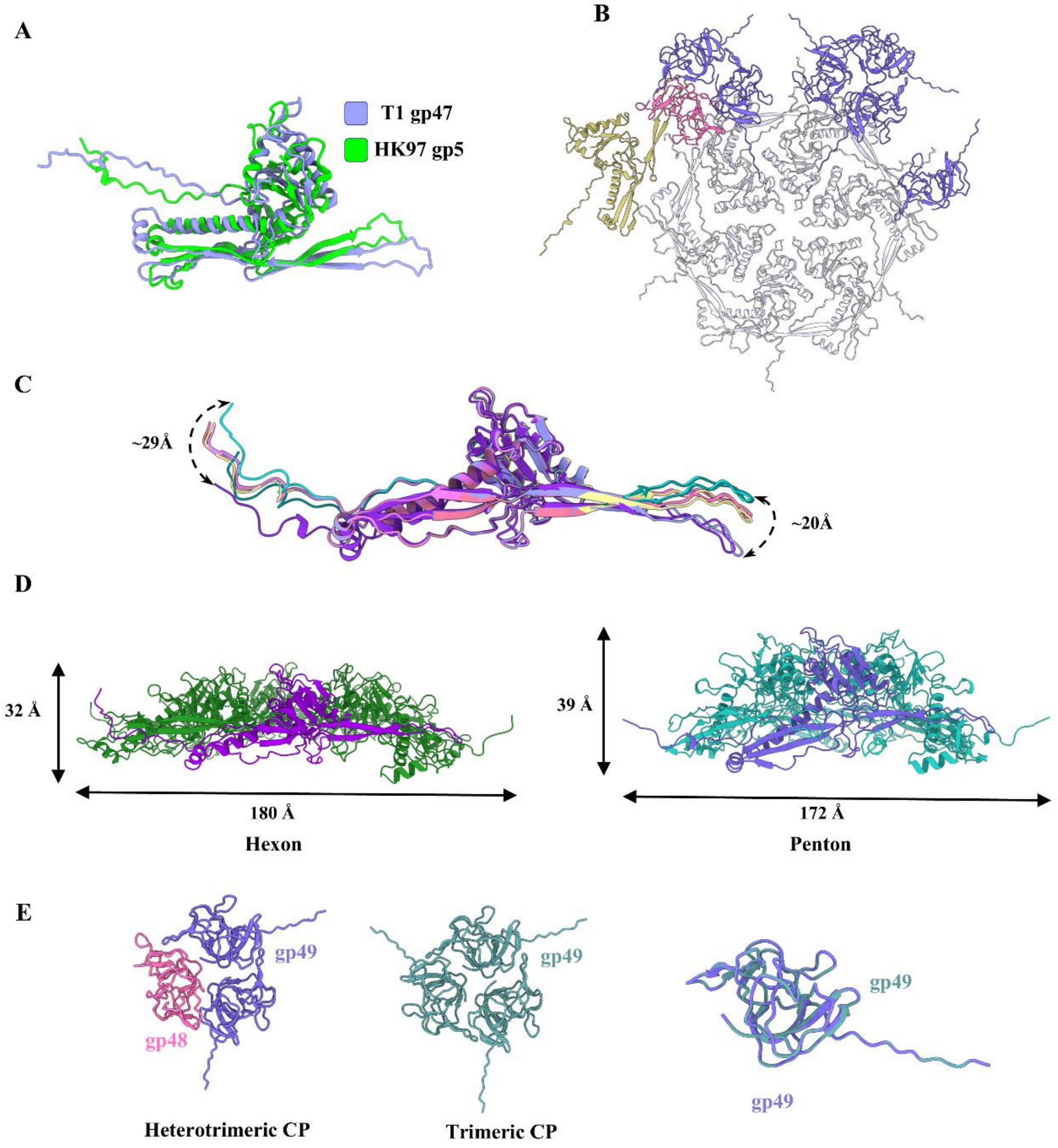

Figure S4. Structures of the MCP and CP of T1. (A) Structural comparison of the ribbon models between the T1 MCP gp47 and the HK97 MCP gp5 (PDB ID: 1OHG). (B) Ribbon models of an asymmetric unit of T1 head. The color coding is identical to that used in Fig. 2A. (C) Superimposition of the seven MCPs in an asymmetric unit, displayed in different colors. (D) Side view of the ribbon models of the hexon (left, green) and the penton (right, light teal green), with the exception of a hexon monomer and a penton monomer, which are colored in dark violet and slate blue, respectively. (E) Structural comparison of gp49 between heterotrimeric CP and trimeric CP.

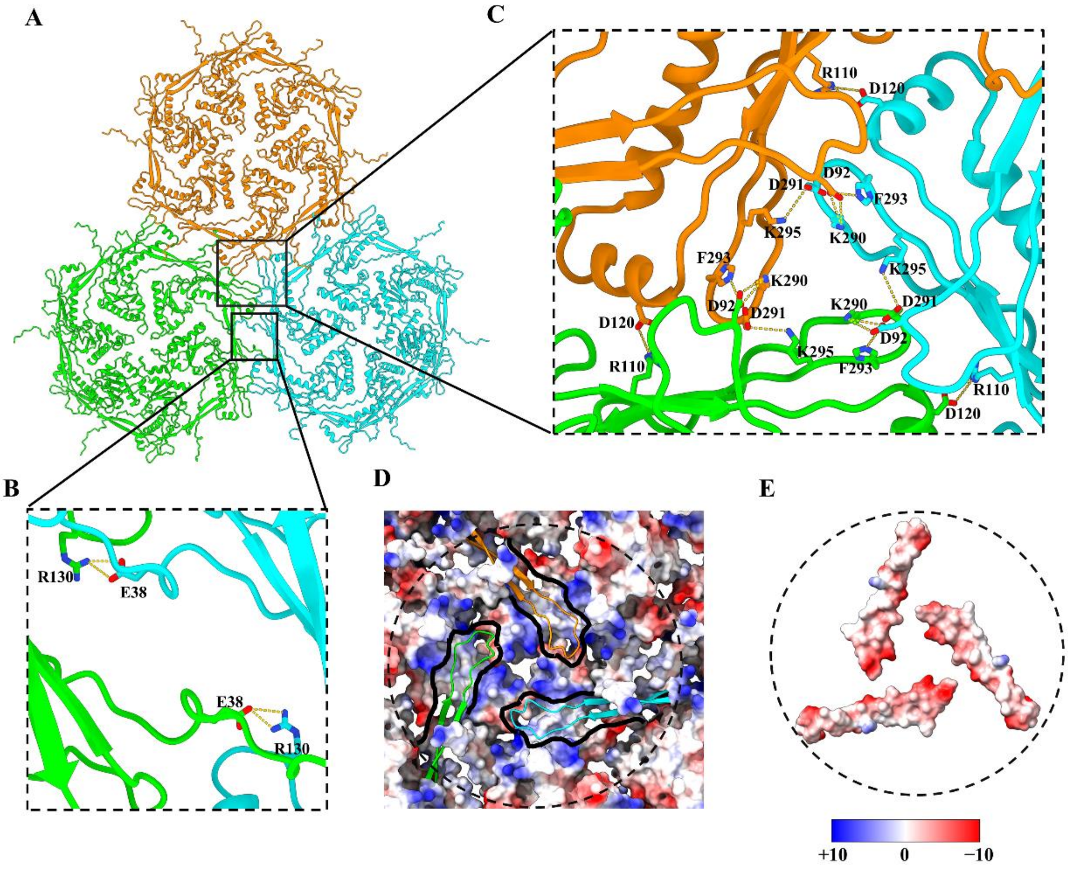

Figure S5. Interactions at the capsomere-capsomere interface around the threefold axis and quasi-threefold axis of the T1 head without CPs. (A) Top view of the inter-capsomeric interactions along the threefold axis without CPs. The three hexons are colored in cyan, orange, and lawn green, respectively. (B, C) Zoomed-in views of the interactions around the twofold axis (B) and the threefold axis (C). H-bonds and salt bridges are indicated by dashed yellow lines. (D) Top view of the electrostatic potential surface around the threefold axis, with the exception of the E-loops shown in ribbon models. (E) Bottom view of the electrostatic potential surface of E-loops coming from the panel D. The electrostatic potential scale is shown in the color bar.

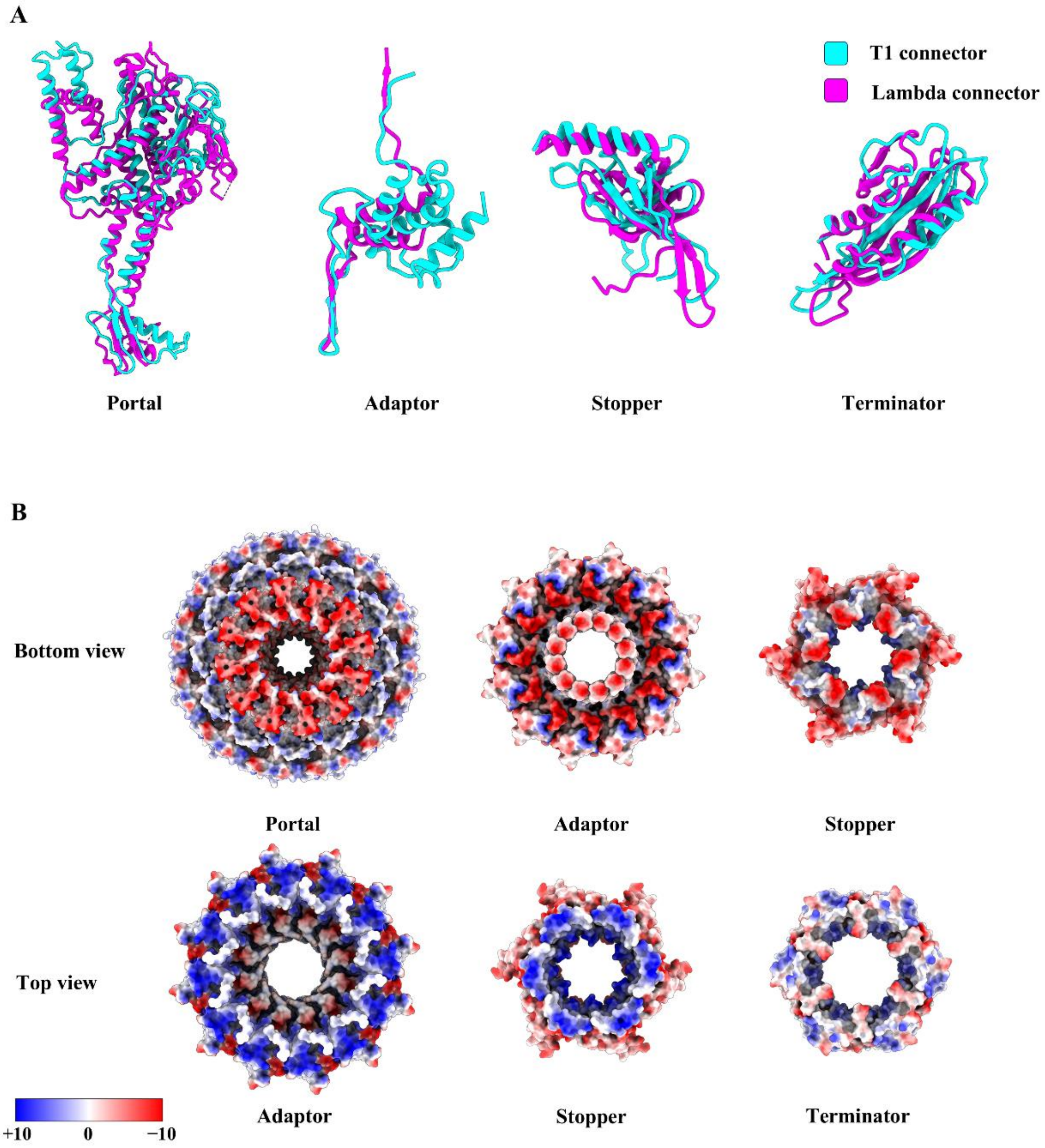

Figure S6. Structures of the connector complex. (A) Structural comparisons of the connector complex between siphophages T1 and Lambda (PDB ID:8k38 and 8k37). (B) Electrostatic potential surfaces of the interacting regions between two adjacent protein components in T1 connector complex. The top columns are oriented toward the tip, whereas the bottom columns are oriented toward the head. The electrostatic potential scale is shown in the color bar.

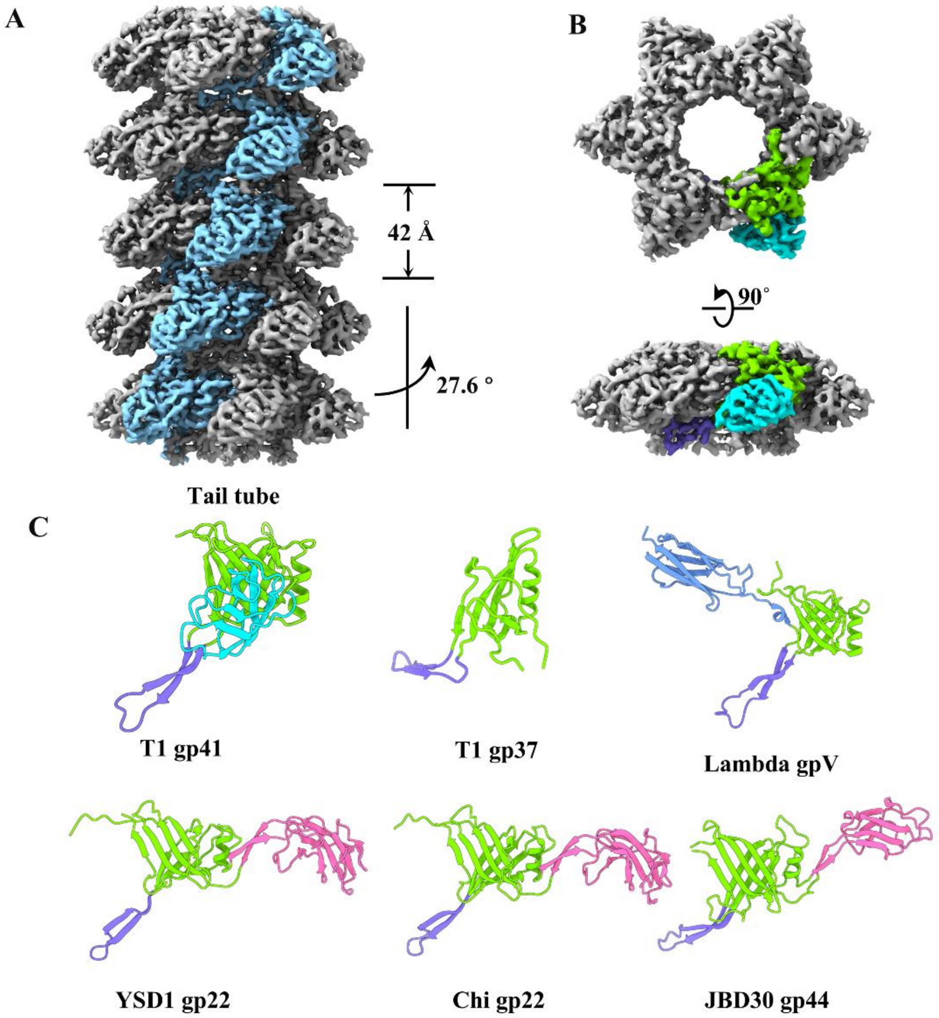

Figure S7. Structural comparison of the tail tube in different siphophages. (A) Side view of the four layers of tail tube. (B) Top and side views of a TTP ring (gray), with the exception of a TTP, which is colored according to its domains. (C) Structural comparisons of the TTP and distal tail protein of T1, as well as TTPs of other siphophages. Gp41 and gp37 in T1, gpV in Lambda (PDB ID: 8iyk), gp22 in YSD1 (PDB ID: 6XGR), gp22 in Chi (PDB ID: 8VJA) and gp44 in JBD30 (PDB ID: 8rk8) are colored according to their domains.

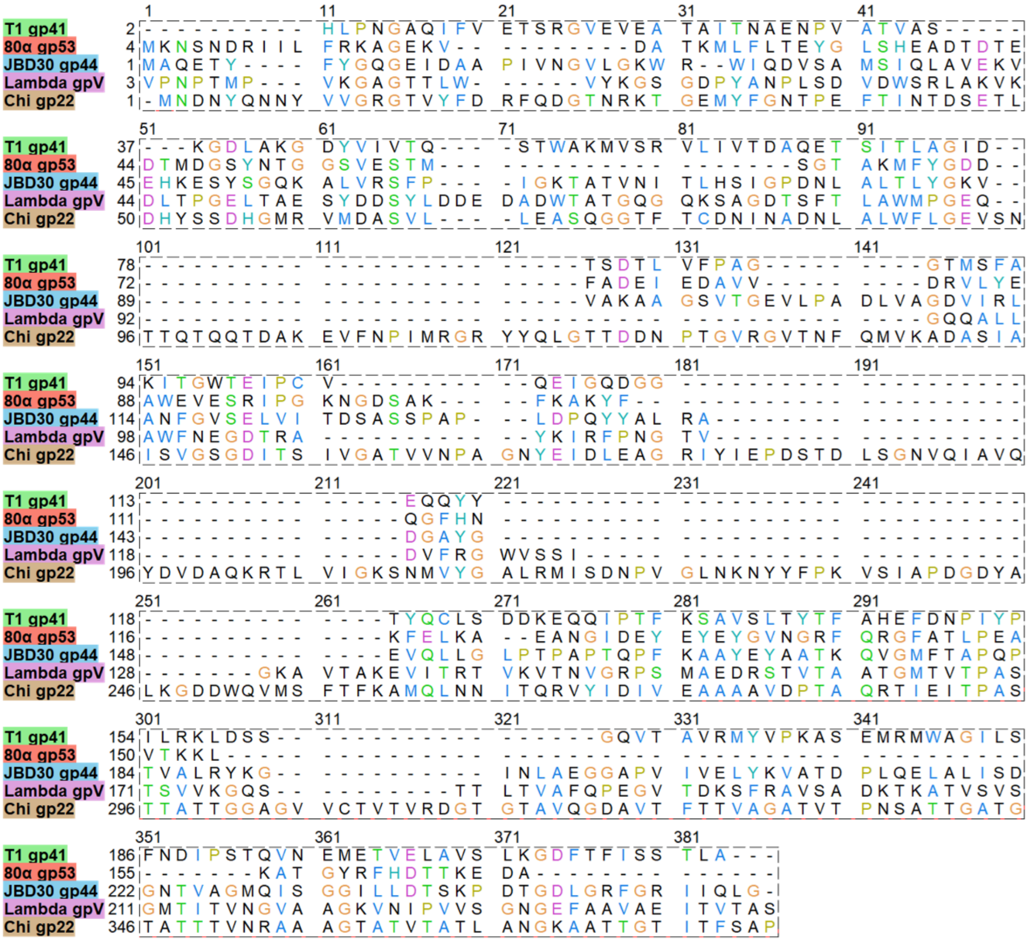

Figure S8. Sequence alignment of the TTPs among phages T1, 80α (PDB ID: 6V8I), JBD30 (PDB ID: 8rk8), Lambda (PDB ID: 8iyk), and Chi (PDB ID: 8VJA). The sequence identity of T1 gp41 compared with 80α gp53, JBD30 gp44, Lambda gpV and Chi gp22 is 9%, 9%, 14% and 7%, respectively, at the amino acid sequence level.

A

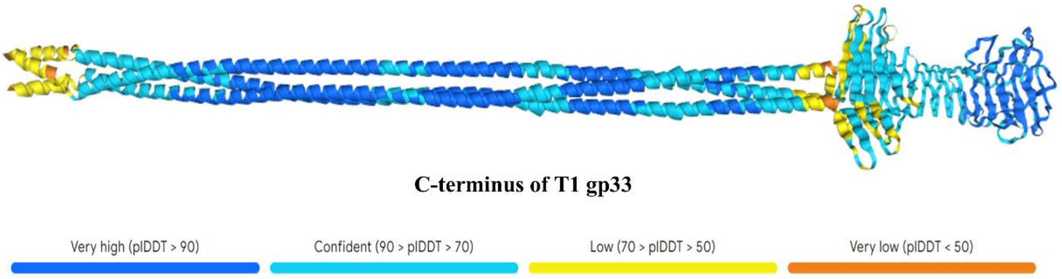

B

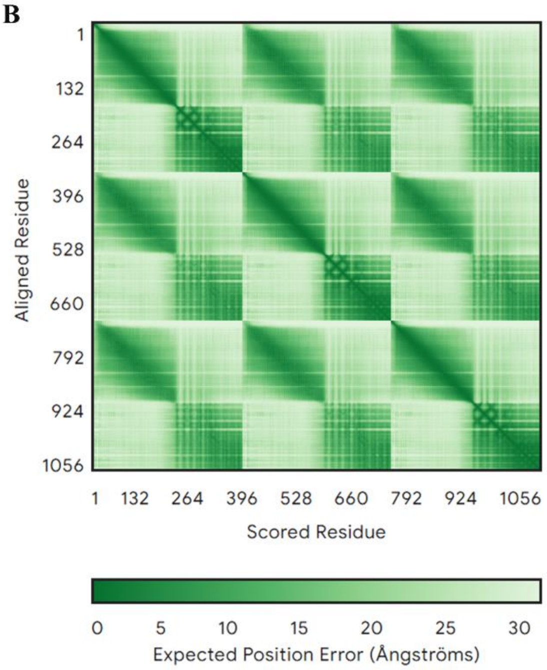

Figure S9. The Predicted Local Distance Difference Test (pLDDT, A) and Predicted Aligned Error (PAE, B) of the C-terminus of T1 gp33, predicted from AlphaFold3.

Table S1. Refinement and model statistics of T1.

Data collection					
Electron microscopy	FEI 300 kV Titan Krios G3i, Gantan K3 camera				
Pixel size (Å)	1.36				
Defocus range	-1.6 to -2.2μm				
Electron exposure	32 e-/Å2				
Total micrographs	3,319				
Local reconstruction					
	Capsid	Portal-adaptor	Neck	Tail tube	Tail tip complex
Symmrtry imposed	C5	C12	C6	C3	C3
Total particles	395,820	99,632	28,865	90,576	27,652
Final particles	320,031	78,790	23,461	34,215	4,467
Resolution(Å)	3.5	3.6	3.6	4.10	4.30
B-factors	150	120	120	200	70
EMDB ID	EMD-62664	EMD-62682	EMD-62698	EMD-62669	EMD-62912
Atomic models refinement/statistics (phenix.real_space_refine ）					
	capsid	Portal-adaptor	Neck	Tail tube	Tail tip complex
PDB ID	9KZJ	9L01	9L0E	9L0F	9L9P
Initial model used (PDB code)	ab-initio	ab-initio	ab-initio	ab-initio	ab-initio
Correlation coefficient (model to map fit)	0.8634	0.7538	0.8242	0.8012	0.7201
Model composition					
Number of chains	14	24	12	6	6
Atoms	24116	48000	18528	14172	10009
Residues	3142	6048	1488	1290	1276
R.m.s. deviations					
Bond lengths	0.003	0.002	0.010	0.003	0.004
Bond angles	0.553	0.511	1.045	0.535	0.768
Validation					
MolProbity score	1.53	1.62	1.69	1.72	2.09
Clash score	5.24	4.58	5.51	5.58	9.74
Rotamer outliers (%)	0.46	0.15	0.39	0.27	0.18
Ramachandran plot (%)					
Favored	96.31	94.37	94.15	93.64	89.02
Allowed	3.34	4.83	5.58	6.05	10.11
Outliers	0.35	0.80	0.27	0.31	0.87

Table S2. The structural similarities and differences of cone-shaped tail tip proteins among Lambda-like siphophages, using Lamba as the reference.

<table>
<tr><th>Phages / Tip proteins</th><th>T1</th><th>Lambda</th><th>T5</th><th>DT57C</th><th>Chi</th></tr>
<tr><td>Distail tail protein</td><td>gp37</td><td>gpM</td><td>pb9</td><td>gp112</td><td>gp26</td></tr>
<tr><td>Hub protein</td><td>gp36</td><td>gpL</td><td rowspan="2">pb3</td><td rowspan="2">gp111</td><td>gp27</td></tr>
<tr><td>Central fiber protein</td><td>gp33</td><td>gpJ</td><td>gp30</td></tr>
<tr><td>Insertion protein</td><td>gp34</td><td>gpI</td><td></td><td></td><td>gp28</td></tr>
<tr><td>Lateral tail fiber</td><td></td><td>stf
tfa</td><td>pb1</td><td>gp108
gp109</td><td></td></tr>
<tr><td rowspan="2">Other proteins</td><td></td><td></td><td>pb4
(Distal fiber protein)</td><td>gp110
(Distal fiber protein)</td><td></td></tr>
<tr><td></td><td></td><td>pb5
(receptor bind protein)</td><td>gp128
(receptor bind protein)</td><td></td></tr>
</table>
